# Isolation of *Bdellovibrio* sp. from soil samples in Mexico and their potential applications in control of pathogens

**DOI:** 10.1002/mbo3.382

**Published:** 2016-06-13

**Authors:** Omotayo Opemipo Oyedara, Erick de Jesus De Luna‐Santillana, Omar Olguin‐Rodriguez, Xianwu Guo, Marco Antonio Mendoza‐Villa, Jorge Luis Menchaca‐Arredondo, Temidayo Oluyomi Elufisan, Javier Alfonso Garza‐Hernandez, Israel Garcia Leon, Mario Alberto Rodriguez‐Perez

**Affiliations:** ^1^ Instituto Politécnico NacionalCentro de Biotecnología GenómicaReynosaTamaulipas88710México; ^2^ Department of Biological SciencesCollege of Science, Engineering and TechnologyFaculty of Basic and Applied ScienceOsun State UniversityOsogboOsun StateNigeria; ^3^ Facultad de Ciencias Físico‐MatemáticasUniversidad Autónoma de Nuevo LeónSan Nicolás de los GarzaNuevo León66455Mexico

**Keywords:** Atomic force microscopy, *Bdellovibrio*, *hit* locus, host‐range

## Abstract

In this study, two strains of *Bdellovibrio* were isolated from soil samples using the culture‐dependent technique and two members of the family *Enterobacteriaceae* (*Klebsiella* sp. and *Salmonella* sp.) as prey. The *Bdellovibrio* strains were bacteriolytic, plaque‐forming, and highly motile gram‐negative bacteria. We identified and confirmed the *Bdellovibrio* strains using microscopy, PCR amplification, and sequencing of the 16S rRNA gene. They were observed to be different strains based on *hit* locus and prey range analyses. Here, the first report on *Bdellovibrio* strains isolated from soil in Mexico corroborates earlier report indicating that populations of *Bdellovibrio* found in soil are heterogeneous thereby the need to identify the various strains.

## Introduction

*Bdellovibrio* and Like Organisms (BALOs) *viz*. genus *Bdellovibrio*,* Bacteriovorax, Peredibacter,* and *Halobacteriovorax* are a group of obligate predatory bacteria that prey upon gram‐negative bacteria for nutrients and reproduction (Snyder et al. 2002; Koval et al. 2015). *Bdellovibrio bacteriovorus*, a member of the BALOs represents one of the most studied predatory bacteria. It is a small (0.2–0.5 *μ*m × 0.5–2.5 *μ*m), uniflagellated motile gram‐negative bacterium that attacks and hydrolyzes cellular constituents of other gram‐negative bacteria, utilizing the derived nutrients for growth and reproduction. The life cycle of *B. bacteriovorus* involves attachment to a suitable gram‐negative prey, loss of flagellum, followed by penetration into prey periplasmic space. At this stage of intraperiplasmic invasion, the prey cell is altered to form a round structure known as bdelloplast. Digestion of prey cellular constituents then takes place and prior to prey lysis, *B. bacteriovorus* undergoes septation to produce progeny that are released to carry out further predation (Stolp and Starr 1963; Chatterjee 2009).

Several publications have indicated *B. bacteriovorus* as a better alternative to treat infections caused by multidrug‐resistant bacteria (Dashiff et al. 2011; Damron and Barbier 2013; Kadouri et al. 2013). It has also been suggested as a biocontrol agent in aquaculture and animal husbandry as against the conventional antibiotics considering the increasing trend of antibiotic resistance among pathogenic bacteria. Kadouri and O'Toole (2005); Monnappa et al. (2014) reported *Bdellovibrio* to successfully degrade or inhibit biofilms produced by both gram‐positive and ‐negative bacteria.

*B. bacteriovorus* is ubiquitous in nature and may be isolated from different sources including plant rhizospheres, freshwater, soil, and gastrointestinal tract of animals (Edao 2000; Jurkevitch et al. 2000; Schwudke et al. 2001; Hobley et al. 2012; Lebba et al. 2013). As mentioned earlier, *B. bacteriovorus* are known to be obligate intracellular predators of gram‐negative bacteria hence it relies solely on the degradation of prey cellular macromolecules and utilization of derived smaller molecular materials for its growth and reproduction. However, *Bdellovibrio* strains that are capable of growing in the absence of prey as well as on nutrient‐rich media mostly referred to as host‐independent *Bdellovibrio* strains have been isolated using current laboratory protocols (Seidler and Starr 1969; Ferguson et al. 2008). Recently, a *B. bacteriovorus* strain Tiberius that is capable of growing simultaneously both in the presence and absence of prey was isolated from River Tiber, Rome (Hobley et al. 2012). The molecular derivation of host‐independent *Bdellovibrio* strains have been linked with a mutation in a genetic *hit* (host interaction) locus coding for proteins that play active role in the attachment and invasion of *Bdellovibrio* into its prey (Cotter and Thomashow 1992). In addition, about 89% of isolated host‐independent *Bdellovibrio* strains have experienced mutation in this locus, 46% demonstrated mutation involving deletion of 42 bp on the *Bd0108* which encodes protein involved in regulation of Type IV pili formation needed for *Bdellovibrio* attachment and invasion (Sockett 2009; Prehna et al. 2014).

The BALOs, in general, exhibit great phylogenetic diversity and their classification have been dynamic in recent years owing to the distinguishing characteristics that exist between the terrestrial (including the freshwater) and marine groups of BALOs. These two groups have been distributed into two different phylogenetic clades based on differences in features such as G+C content, salt tolerance and prey range (Baer et al. 2000). Consequently, the marine BALOs were separated from the Genus *Bdellovibrio* and renamed *Bacteriovorax*. In fact, the nomenclature of BALOs associated with marine habitat was recently changed from *Bacteriovorax* to *Halobacteriovorax* (Koval et al. 2015). In the soil habitat, particularly, *Bdellovibrio* has been described to represent a heterogenous community of predatory bacteria that utilize wide range of gram‐negative bacteria as prey (Jurkevitch et al. 2000).

Here, the first report in Mexico on molecular characterization of two *Bdellovibrio* strains isolated from soil using culture‐dependent technique is presented. The two *Bdellovibrio* strains were found at a neighborhood in the city of Reynosa (a border city to McAllen, Texas in the USA), Tamaulipas State and were characterized using 16S rRNA gene sequencing, *hit* locus PCR amplification and prey range analyses.

## Materials and Methods

### Sample collection

Soil samples were collected at two different sites on a plot of land (26.069678N′, −98.313108W′ and 26.069446N′,−98.312902W′) within the Center for Genomic Biotechnology, National Polytechnic Institute [IPN as in Spanish] located in the city of Reynosa, Mexico. The soil samples were collected with a clean hand trowel after removing about 20–25 mm top of soil and put into a sterile polythene bag. The soil samples were immediately transported to the laboratory for analysis and isolation of *Bdellovibrio* strains.

### Determination of soil pH and electrical conductivity

Soil sample was suspended in 100 mL of deionized water and stirred for 5 min. The suspension was left on the bench overnight (OV) and stirred again afterward. It was further left for 15 min and the liquid portion was transferred into a clean beaker. The pH and electrical conductivity of the soil sample was then measured using HI 991300^TM^ pH/EC/TDS/Temperature meters (Hanna instruments, Woonsocket, Rhode Island, USA) (Chaudhari et al. 2014).

### Host Preparation

The bacterial preys used for preliminary isolation of the *Bdellovibrio* strains were *Klebsiella* sp. and *Salmonella* sp. donated by Biotechnology Institute (Autonomous University of Nuevo Leon [UANL as in Spanish] located in San Nicolás, de los Garza, Nuevo León, Mexico) and Center for Genomic Biotechnology, respectively. The different preys were cultured in Luria Bertani (LB) broth for 24 h and 0.4 mL was mixed with 0.1 mL of filtrate for the double layer agar plating technique. For the liquid culture medium analysis, the bacterial preys were cultured in LB broth for 24 h, harvested by centrifugation, washed and resuspended in 25 mmol/L HEPES buffer (4‐[2‐hydroxyethyl]‐1‐piperazineethanesulfonic acid) containing 3 mmol/L CaCl_2._ 2H_2_O and 2 mmol/L MgCl_2._ 6H_2_O (pH 7.4).

### Isolation and determination of lytic activity of *Bdellovibrio* strains

Soil samples were suspended in 100 mL of HEPES buffer and shaken for 1 h at 200 rpm. The suspension was centrifuged at 1,800g for 5 min. The resulting supernatant was filtered serially using a 0.8 *μ*m and 0.45 *μ*m syringe filter (MF‐Millipore^TM^ Membrane, Merck Millipore Ltd, Tullagreen, Carrigtwohill, Co. cork, Ireland). The filtrate was serially diluted and plated on dilute nutrient broth (DNB) agar (0.08% nutrient broth amended with 3 mmol/L MgCl_2_. 6H_2_O, 2 mmol/L CaCl_2_. 2H_2_O, 0.6% agar for top agar, and 1.9% for bottom agar [pH 7.2]) using double‐layer agar plating technique (Stolp and Starr 1963; Jurkevitch 2012). The plates were incubated at 30°C and plaque development on the DNB agar was monitored for 7 days. Plaques which emerge on DNB agar between 48 to 72 h and progressively increase in size were taken to be potential *Bdellovibrio* plaques. The purification of plaques obtained was done by single‐plaque isolation technique (Jurkevitch 2012).

For determination of lytic activity, a single pure plaque was cut into 5 mL suspension of bacterial prey cells already washed with HEPES buffer using pipette tip cut with sterile scalpel. The culture was incubated at 30°C and monitored for prey lysis. Microscopic examination was carried out alongside to determine the presence of highly motile *Bdellovibrio* strains. The clear HEPES buffer culture obtained from the prey lysis was centrifuged three times at 2,800g for 15 min (Schwudke et al. 2001). The resultant supernatant (the lysate) was further used to inoculate large volume of appropriate bacterial prey already washed with HEPES buffer. The initial optical density of the host was read at 600 nm using optizen POP spectrophotometer (Mecasys Co., Ltd, Daejeon, Korea) and the progressive reduction in turbidity with time was monitored to determine the lytic activity of the *Bdellovibrio* strains. The pure *Bdellovibrio* strains obtained through separation from prey by successive filtration as described earlier and concentrated at 27,000*g* as well as lysates were stored in sterile glycerol at 80°C for further study.

### Microscopic identification of isolated *Bdellovibrio* spp using atomic force microscope

The lysate was observed using Olympus U‐TVO.35XC‐2 light microscope (T2 Tokyo, Japan) for characteristic high motility of *Bdellovibrio* strains. The atomic force microscopy study (AFM) was done at the Department of Cellular Biology and Genetics (UANL). Briefly, 10 *μ*L of lysate from prey and *Bdellovibrio* strains cocultured in HEPES buffer was deposited on cleaved mica and allowed to air‐dry. The bacteria samples were observed using an NT‐MDT NTEGRA Prima AFM at room temperature, with a RTESPA probe (Bruker corporation, Beijing China) of spring constant k = 40 N/m in intermittent contact mode. Images of height, deflection, and phase were obtained; 20 × 20, 10 × 10, and 5 × 5 *μ*m^2^ image sizes were captured systematically for each sample at three different regions at least. They were analyzed with WSXM software to observe the morphological aspect of the bacteria (Nunez et al. 2003; Horcas et al. 2007).

### Amplification of 16S rRNA gene and *hit* locus by PCR

For the detection of *Bdellovibrio* strains, a clear lysate was centrifuged three times at 2,800*g* for 15 min to remove residual prey cells (Schwudke et al. 2001). The final resultant supernatant was centrifuged at 27,000*g* for 20 min. DNA extraction was done using Wizard^®^ Genomic DNA purification kit (Madison, Wisconsin, USA) according to manufacturer's instructions. The 16S rRNA gene was amplified using the Ref.‐fwd. primer; 5′ TTTCGCTCTAAGATGAGTCCGCGT‐3′ and Ref.‐rev. primer; 5′‐TTCGCCTCCGGTATTCCTGTTGAT‐3′ (Van Essche et al. 2009) that amplified a 492 bp fragment of the 16S rRNA gene. The presence of few residual prey bacterial cells even after centrifugation was observed in this study and reported by Parker and Grove (1970); Schwudke et al. (2001).

Therefore for purification and amplification of considerable length of the 16S rRNA gene, large volume of lysates from prey–predator coculture was centrifuged twice at 1,800g for 15 min at 4°C. The supernatant was serially filtered through 0.80 *μ*m, 0.65 *μ*m, and 0.45 *μ*m syringe filter. At each stage of filtration, microscopic examination to observe the presence of fast moving *Bdellovibrio* as well as cultivation of the filtrate on LB agar using spread plate technique to ascertain total elimination of prey bacterial cell was done. In addition, double‐layer agar plating technique was carried out at each stage of filtration to ascertain the presence of plaque‐forming *Bdellovibrio* strains. Finally, the filtrate obtained was centrifuged at 27,000*g* for 20 min. The genomic DNA was extracted as previously described above and the 16S rRNA gene was amplified with the Bdello.‐fwd. primer; 5′ AGAGTTTGATTCTGGCTCAGA‐3′ and Bdello.‐rev. primer; 5′‐AGGTGATCCAGCCGCAGGTTC‐3′ which amplified a 1493 bp fragment of the 16S rRNA gene in an *in silico* PCR using an online tool available on the website of University of the Basque Country (http://insilico.ehu.es/PCR/Amplify) with the genomes of different species and strains of *Bdellovibrio* listed in the online software serving as template. And for the amplification *hit* locus, the following hit‐fwd. primer; TCTAGACAGATGGGATTACTG and hit‐rev. primer; GAATTCTGGCATCAACAGC which amplified a 959 bp were used.

PCR amplification was performed in 0.5 mL Eppendorf tube using the following conditions: Predenaturation at 95°C for 5 min, 30 cycles of denaturation at 95°C for 30 sec, annealing at 60°C (ref.‐fwd., ref.‐rev., hit‐fwd., and hit‐rev. primers) and 62°C (Bdello.‐fwd. and Bdello.‐rev. primers) for 30 sec, extension at 72°C for 40 sec, and final extension at 72°C for 10 min. The amplified product was analyzed by 1% agarose gel electrophoresis.

### Cloning, DNA sequencing, and analysis

The amplified and purified 16S rRNA fragment was ligated into PGEM^®^‐T vector (Promega^®^, Madison, Wisconsin, USA) and transformed into *Escherichia coli* DH5*α* competent cells. The cloning procedure was performed according to manufacturer's instructions. Plasmids from positive clones were sequenced by Eurofins MWG Operon© LLC company (www.operon.com; Huntsville, Alabama). The obtained sequences were analyzed using Lasergene program Seqman^®^ software (DNAstar Inc., Madison, Wisconsin, USA) and subjected to similarity searches against 16S rRNA gene sequences retrieved from ribosomal database project (RDP) and NCBI databases. A neighbor joining (NJ) analysis was carried out using the pairwise distances (Kimura two‐parameter model) metric to recover their clustering pattern. Bootstrap values were calculated to test the robustness of interior node support and were obtained by conducting 1000 pseudoreplicates using MEGA© 6.0 software (Tamura et al. 2013).

### Prey range analysis

The prey range analysis was carried out using double layer agar plating technique as described above with 36 bacterial isolates including 21 referenced bacterial isolates obtained from the National Collection of Microbial Strains and Cell Cultures at the, Research Center for Advanced Studies (CINVESTAV as in Spanish) of IPN located in México City, México and 15 laboratory bacterial strains obtained from Biotechnology Institute of UANL and Center for Genomic Biotechnology of IPN. The reference bacterial strains were cultured as recommended by the culture collection center while the laboratory strains were cultured in LB medium for 24 h. The bacterial preys used in isolation of the different *Bdellovibrio* strains (*Klebsiella* sp. and *Salmonella* sp.) served as the positive control while bacterial prey without *Bdellovibrio* and filtrates containing *Bdellovibrio* without prey served as negative control. The experiment was carried out in duplicates and plaque formation was monitored for at least 7 days.

### Statistical analysis

Each experiment and control for determination of lytic activity was carried out in triplicates with *Klebsiella* sp. and *Salmonella* sp. preys suspended in HEPES buffer without *Bdellovibrio* strains serving as control. The transformation of mean values of optical density was done using square root for variance normalization. The transformed mean values of optical density (on y‐axis) were plotted against time (on x‐axis). Statistical analysis was performed using Excel© for Windows©, 2013 (Microsoft, Redmond, Washington, USA). Student's *t*‐test was used to compare the mean values of the two groups and *P* < 0.05 was used as indicator of significant difference.

## Results and Discussion

### Isolation and determination of lytic activity of *Bdellovibrio* strains

**Figure 1:**
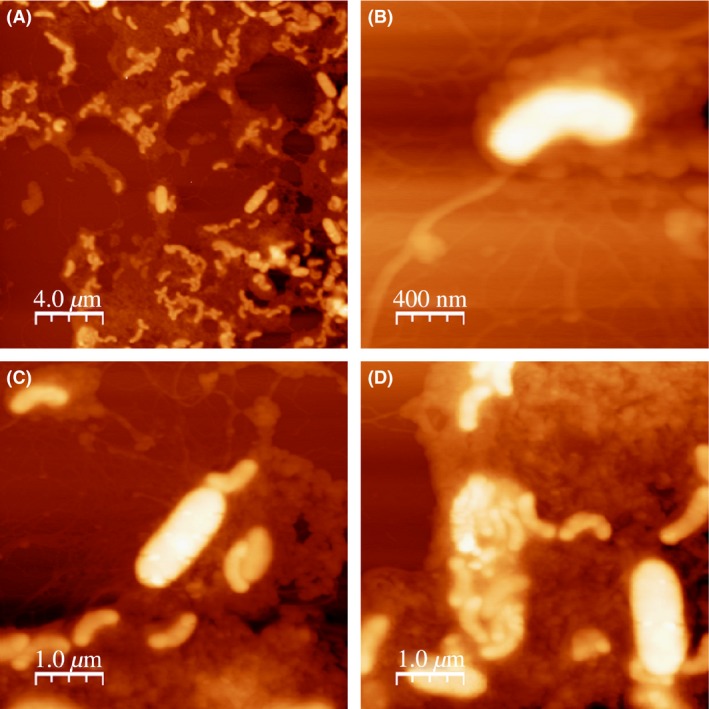
Microscopic identification of isolated *Bdellovibrio* strain SSB218315 using atomic force microscope. (A) Image showing coculture of long rod‐shaped *Salmonella* sp. and coma‐shaped *Bdellovibrio* strain SSB218315. (B) Image showing coma‐shaped *Bdellovibrio* strain SSB218315 only. (C) Image showing attachment of *Bdellovibrio* strain SSB218315 to *Salmonella* sp. (D) Image showing clustering of *Bdellovibrio* strain SSB218315 in a way probably suggesting host lysis.

Two different strains of *Bdellovibrio* designated SKB1291214 and SSB218315 were isolated from soil under a banana (*Musa paradisiaca* L) plant at a neighborhood from the city of Reynosa, Mexico (IPN), using DNB agar with plaque development observed within 2–7 days on *Klebsiella* sp. and *Salmonella* sp. preys, respectively. The plaques formed by isolated *Bdellovibrio* strains were irregular and expanded for many days as previously described by Stolp and Starr (1963) and Jurkevitch (2012). The soil samples were moist dark‐brown loamy soil with pH of 7.3 and electrical conductivity of 0.20 mScm^−1^ suggesting their good agricultural value. The light microscopy examination revealed the isolated *Bdellovibrio* strains as highly motile, rod (comma) shaped gram‐negative bacteria. Further examination of SSB218315 under atomic force microscopy revealed clearly two kinds of bacteria, the small comma‐shaped *Bdellovibrio* (predator) and the bigger long rod‐shaped *Salmonella* (prey). One of the images also showed the attachment of *Bdellovibrio* to the prey and another one showed the clustering of the *Bdellovibrio* strains in a fashion that perhaps depict a prey cell that was just lysed (Fig. 1, panels A, B, C, and D).

*Bdellovibrio* spp. have been reported to be frequently encountered in soil representing about 80% of all BALOs in soil community (Fulthorpe et al. 2008). However, the ecological role of *Bdellovibrio* spp. in the different niche where they are encountered is not well understood, perhaps serving as an “ecological balancer” as described by Lebba et al. (2014). This study further supports the use of DNB agar in double agar plating technique as an effective method of isolating *Bdellovibrio* strains from the soil. In addition, the prey choice can also be one of the determining factors for the successful isolation of *Bdellovibrio* strains; preferably, gram‐negative bacteria that differ from *Bdellovibrio* strains most especially in size and motility could be considered for successfully isolation of *Bdellovibrio* strains. This will allow easy differentiation of *Bdellovibrio* strains from their prey under the microscope.

**Figure 2:**
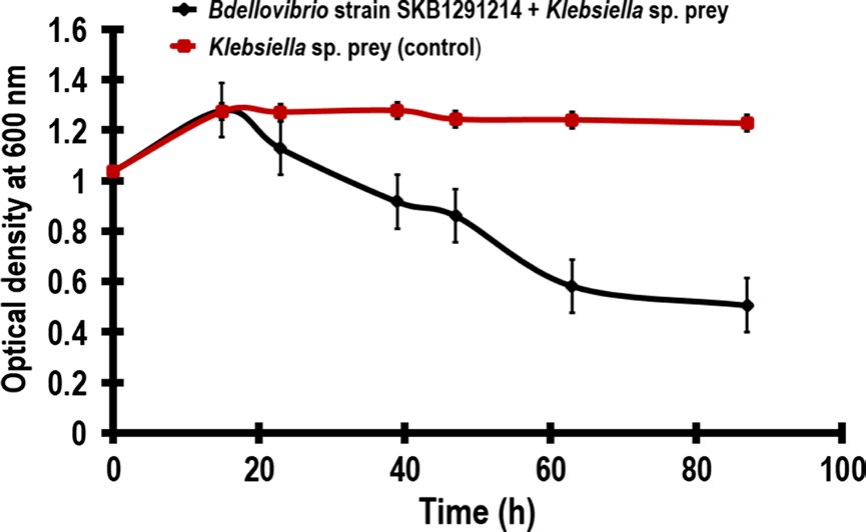
The lytic activity of *Bdellovibrio* strain SKB1291214 (1.22 × 10^6^ PFUmL^−1^) when *Klebsiella* sp. was infected. The graph shows mean values of optical density (y‐axis) against time (x‐axis) with error bars showing standard error. *R* ^*2*^ = 0.81 (for experimental) and 0.16 (for control). *t* = 0.01, *P* < 0.05.

**Figure 3:**
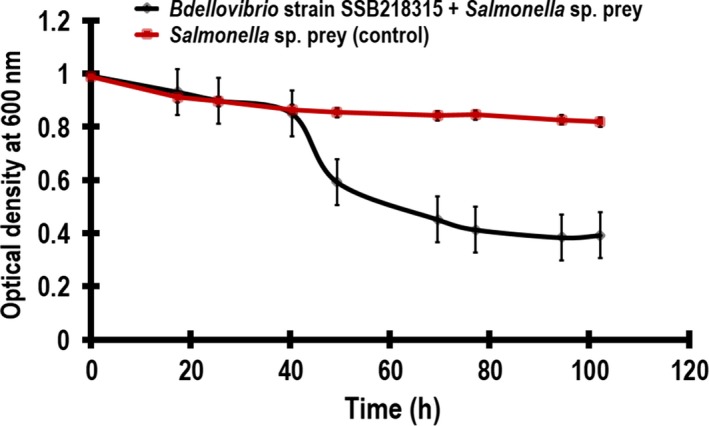
The lytic activity of *Bdellovibrio* strain SSB218315 (8.62 × 105 PFUmL^−1^) when *Salmonella* sp. was infected. The graph shows mean values of optical density (y‐axis) against time (x‐axis) with error bars showing standard error. *R* ^*2* ^= 0.91 (for experimental) and 0.83 (for control). *t* = 0.02, *P* < 0.05.

*Bdellovibrio* strain SKB1291214 was able to attack and lyse the *Klebsiella* sp. in liquid medium. This was evident with the reduction in optical density [(1.07–0.26) before statistical square root transformation] within 72 h (Fig. 2). Similarly, *Bdellovibrio* strains SSB218315 lysed the *Salmonella* sp. in liquid medium reducing the optical density from 0.98 to 0.15 [values obtained before statistical square root transformation (Fig. 3)]. The statistical analysis of the mean optical density values of the experimental (0.90 ± 0.28 SD) when *Klebsiella* sp. was infected with *Bdellovibrio* strain SKB1291214 was significantly different in comparison with the mean optical density values of the control (1.22 ± 0.09 SD) as determined by student's *t*‐test (*t* = 0.007, *P* < 0.05). In a similar way, there was significant difference (*t* = 0.01, *P* < 0.05) using Student's *t*‐test to compare mean optical density values of the experimental (0.65 ± 0.26 SD) and control (0.87 ± 0.05 SD) when *Salmonella* sp. was infected with *Bdellovibrio* strain SSB218315. The ability of *Bdellovibrio* strains SKB1291214 and SSB218315 to lyse *Klebsiella* sp. and *Salmonella* sp. suggested the possibility of using these *Bdellovibrio* strains to control pathogenic strains of these study preys.

### Molecular identification of *Bdellovibrio* spp isolated using 16S rRNA and *hit* locus

**Figure 4:**
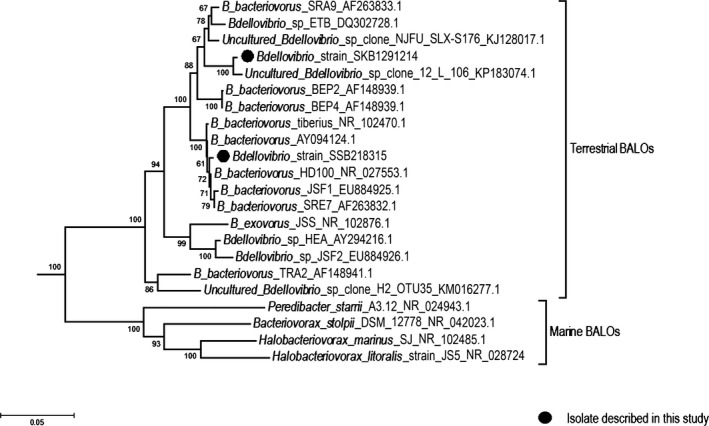
Phylogenetic tree of partial 16S rRNA gene sequences constructed with neighbor‐joining algorithm using the pairwise distances (Kimura two‐parameter model) metric to recover their clustering pattern. The numbers at each node represents bootstrap values for 1000 replicates. *Thermotoga maritima* 16S rRNA gene sequence was used as an external group.

*Bdellovibrio* SKB1291214 and SSB218315 strains gave PCR products for the 16S rRNA gene using the aforementioned primers similar to the fragments of 492 bp and 1493 bp amplified with reference strain *B. bacteriovorus* HD100 donated by the Department of Plant Pathology and Microbiology of the Hebrew University of Jerusalem, Israel serving as positive control. The blast search result of the sequenced 16S rRNA gene from RDP showed that the two isolated strains belonged to class *δ proteobacteria*, family *Bdellovibrionaceae,* and genus *Bdellovibrio*. The NJ tree constructed using the 16S rRNA gene (Fig. 4) showed that *Bdellovibrio* SKB1291214 and SSB218315 strains clustered with other group of *Bdellovibrionaceae* (*δ ‐proteobacteria*) but different from each other. *Bdellovibrio* SKB1291214 strains show similarity with an uncultured *Bdellovibrio* sp. clone 12L 106 (accession number KP183074.1) with their sequences exhibiting 99% identity. It further clustered with two soil associated strains of *Bdellovibrio* spp. namely *Bdellovibrio* sp. ETB (accession number DQ302728.1) and *B. bacteriovorus* SRA9 (accession number AF263833.1). Meanwhile, *Bdellovibrio* SSB218315 strains clustered with groups of reported soil‐associated *Bdellovibrio* spp. including the first reported *Bdellovibrio bacteriovorus* HD100 (accession number NR027553.1) as well as *Bdellovibrio bacteriovous* strain Tiberius (accession number NR102470.1) isolated from fresh water aquatic environment.

**Figure 5:**
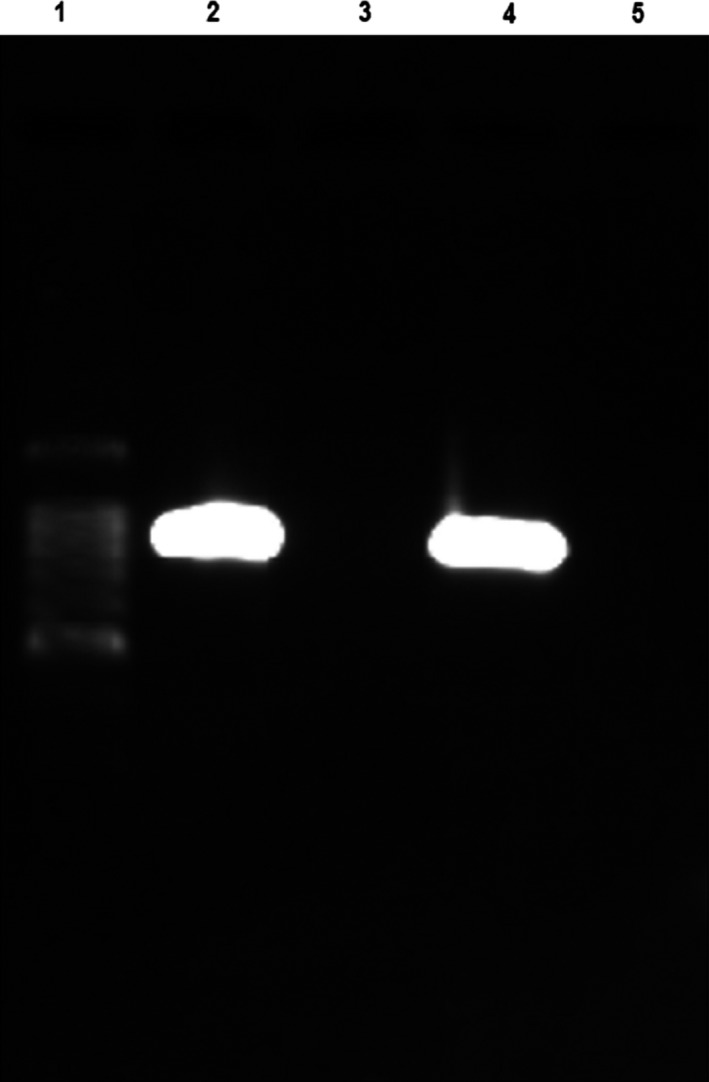
Amplification of 959 bp fragment of the *hit* locus.(1) 100 bp Ladder, (2) HD100‐control, (3)SKB1291214, (4) SSB218315, (5) Distilled water.

The *hit* locus was successfully amplified in *Bdellovibrio* strain SSB218315 strains but not in *Bdellovibrio* SKB1291214 strains (Fig. 5). Sequencing and analysis of the amplified product from *Bdellovibrio* strain SSB218315 further confirmed it to be *hit* locus. The blast analysis showed the two study *Bdellovibrio* strains exhibiting 97% identity with two rhizosphere‐derived *Bdellovibrio bacteriovorus* strains BEP2 and BRP4 reported by Jurkevitch et al. (2000). However, these two strains clustered more closely to *Bdellovibrio* strain SKB1291214 than SSB218315 on the NJ tree. And interestingly, *hit* locus was not successfully amplified in this two reported strains (*Bdellovibrio bacteriovorus* strains BEP2 and BRP4) when PCR technique was used. This is an indication that *Bdellovibrio* strain SKB1291214 may possibly be a plant rhizosphere‐associated strain. Furthermore, since the *hit* locus has been proposed to be restricted to *B. bacteriovorus* (Schwudke et al. 2001), electron microscopy may provide more information on *Bdellovibrio* strain SKB1291214. Perhaps, it may be using different mechanism of action for its predatory activities similar to an epibiotic *Bdellovibrio exovorus* in which the *hit* locus has not been amplified as reported by Koval et al. (2013).

In addition, the distant relationship that has been reported to exist between the marine; family *Bacteriovoraceae* and terrestrial; family *Bdellovibrionaceae* (including the freshwater) groups of “*Bdellovibrio* and like organisms” (BALOs) as reported by Baer et al. (2000) and Jurkevitch et al. (2000) can also be inferred from the NJ tree. These two groups of BALOs were initially grouped together as *Bdellovibrionaceae* but later separated into two groups based on differences in the characteristics including variations in G+C content, prey preference, and response to salinity.

The 16S rRNA gene sequence data obtained in this study was processed with online software Decipher^®^ (Wright et al. 2012) to check chimeras and have been submitted to GenBank Databases under accession numbers KT852580.1 and KT807464.1 for *Bdellovibrio* strain SSB218315 and *Bdellovibrio* strain SKB1291214, respectively. Details of data submission can be found at GenBank: www.ncbi/nlm.nih.gov.

### Prey range analysis

*Bdellovibrio* strain SKB1291214 was able to form plaque with 13 of 36 (36.11%) bacterial isolates considered for prey range analysis while *Bdellovibrio* strain SSB218315 was able to prey upon 22 (61.11%) bacterial isolates. None of the *Bdellovibrio* strains formed plaque on the six gram‐positive bacteria viz. genera *Staphylococcus* and *Bacillus* considered in this study (Table 1). Most of the prey considered belongs to the phylum *γ‐proteobacteria* with two *α‐proteobacteria* (*Rhizobium leguminosarum* and *Agrobacterium tumefaciens*) and only one *β‐proteobacteria* (*Alcaligenes* sp). *Bdellovibrio* strain SSB218315 preyed upon all the bacteria belonging to family *Enterobacteriaceae* except *Citrobacter freundii*. However, *Bdellovibrio* strain SKB1291214 on the contrary was able to utilize this *Citrobacter freundii* as prey.

**Table 1:** Prey range analysis of *Bdellovibrio* strains SKB1291214 and SSB218315 on thirty‐six (36) bacterial isolates

S/No	Bacterial prey	SKB1291214 (36.11%)	SSB218315 (61.11%)
1	*Klebsiella oxytoca* B‐968 (ATCC 13182)^a^	+	+
2	*Klebsiella pneumoniae subspecie pneumonia* B‐969 (ATCC 13883)^a^	+	+
3	d *Klebsiella* sp (Laboratory strain)^b^	+	+
4	*Salmonella enterica* subsp. Enterica serovar typhi CDBB‐B‐1101 (ATCC 7251)^a^	+	+
5	e *Salmonella* sp A (Laboratory strain)^c^	+	+
6	*Salmonella* sp B (Laboratory strain)^c^	+	+
7	*Salmonella* sp D (Laboratory strain)^c^	+	+
8	*Pseudomonas aeruginosa* CDBB‐B‐1021 (ATCC 27853)^a^	−	+
9	*Pseudomonas putida* CDBB‐B‐93 (ATCC 795)^a^	−	+
10	*Pseudomonas fluorescens CDBB‐B‐*1243 (ATCC 13525)^a^	−	+
11	*Enterobacter aerogenes* CDBB B‐958 (ATCC 13048)^a^	+	+
12	*Serratia marcescens* CDBB‐B‐1014 (ATCC 14756)^a^	−	+
13	*Vibrio cholerae* CDBB‐B 1159 (ATCC 39540)^a^	−	+
14	*Staphylococcus aureus* subsp *aureus* CDBB‐B‐1001 (ATCC 6538)^a^	−	−
15	*Staphylococcus aureus* AR2 (Laboratory strain)^c^	−	−
16	*Staphylococcus aureus* B (Laboratory strain)^c^	−	−
17	*Staphylococcus epidermidis* CDBB‐B‐1012 (ATCC 12228)^a^	−	−
18	*Bacillus thuringiensis* CDBB‐B‐26 (ATCC 13366)^a^	−	−
19	*Bacillus cereus* CDBB‐B‐949 (ATCC 6464)^a^	−	−
20	*Citrobacter freundii* CDBB‐B‐955 (ATCC 8090)^a^	+	−
21	*Alcaligenes* sp. CDBB‐B‐17 (ATCC 27066)^a^	−	+
22	*Escherichia coli* CDBB‐B‐1107 (ATCC 8739)^a^	−	+
23	*Escherichia coli* 5A (Laboratory strain)^c^	+	+
24	*Escherichia coli* 5B (Laboratory strain)^c^	+	+
25	*Escherichia coli* 2 (Laboratory strain)^c^	−	+
26	*Escherichia coli* 4 (Laboratory strain)^c^	+	+
27	*Escherichia coli* 3B (Laboratory strain)^c^	+	+
28	*Escherichia coli* DH5*α* a	−	−
29	*Proteus mirabilis* CDBB‐B‐1343 (ATCC 21100)^a^	−	+
30	*Stenotrophomonas sp* C18414^c^	−	−
31	*Stenotrophomonas sp* A23414^c^	−	−
32	*Stenotrophomonas sp* CAZ^c^	−	−
33	*Pseudomonas sp* DTB^c^	−	+
34	*Rhizobium leguminosarum* (CDBB‐B‐1885)^a^	−	−
35	*Agrobacterium tumefaciens* (CDBB‐B‐1042)^a^	−	−
36	*Pseudomonas syringae patovar aceris* (ATCC 10853)^a^	−	−
	*N*	13	22

*N*: number of bacteria preyed upon.

a Reference bacterial isolates obtained from National Collection of Microbial Strains and Cell Culture of CINVESTAV.

b Laboratory bacterial strains obtained from Biotechnology Institute of UANL.

c Laboratory bacterial strains obtained from Center for Genomic Biotechnology (IPN).

d Positive control for *Bdellovibrio* strain SKB1291214.

e Positive control for *Bdellovibrio* strain SSB218315.

Furthermore, *Alcaligens* sp. belonging to the phylum *β‐proteobacteria* was preyed upon only by *Bdellovibrio* strain SSB218315. *Stenotrophomonas* spp. (family *Xanthomonadaceae*) and *Rhizobium leguminosarum were* not preyed upon by the two isolated *Bdellovibrio* strains.

*Bdellovibrio* strains have wide prey range but limited to gram‐negative bacteria. Here, *Bdellovibrio* strain SSB218315 was observed to utilize more bacterial prey compared to *Bdellovibrio* strain SKB1291214. This could be expected because preferential predation has been reported in BALOs (Li et al. 2011). However, the two strains exhibited some preference for the bacterial isolates belonging to the family *Enterobacteriaceae* and coupled with the fact that *Bdellovibrio* strains have been isolated from guts of mammals (Schwudke et al. 2001; Lebba et al. 2013), they could be used to stabilize intestinal bacterial flora perhaps as probiotics. *Rhizobium leguminosarum* lives symbiotically with root of leguminous plants helping in nitrogen fixation which in turn aid plant growth. The inability of the two strains of *Bdellovibrio* to prey upon *Rhizobium leguminosarum* suggests the possibility of using the two bacteria synergistically to help plant growth.

One of the setbacks that could limit the application of *Bdellovibrio* strains is their inability to attack or utilize gram‐positive bacteria as prey as equally observed in this study with the inability of the study strains to form plaque on all the gram‐positive bacteria considered for the prey range analysis. However, the ability of *Bdellovibrio bacteriovorus* HD100 to survive in the presence of *Staphylococcus aureus* using epibiotic mode of attack has been reported (Lebba et al. 2014).

In summary, the two *Bdellovibrio* strains isolated from soil exhibited the ability to prey upon different types of gram‐negative bacteria and this attribute could be considered for future use in the control of pathogenic gram‐negative bacteria. The differences observed between the two strains of *Bdellovibrio* isolated here with respect to amplification of *hit* locus and prey range further support Jurkevitch et al. (2000) report that population of *Bdellovibrio* found in the soil is made up of heterogenous groups. Therefore, this suggests the need for further characterization and classification of soil‐associated *Bdellovibrio* for possibility of grouping them into different subgroups (strains). Finally, with the paucity of information on BALOs research in Mexico, this work is expected to pave way for basic line of research in BALOs with ultimate goal of utilizing them for biotechnological applications.

## Conflict of Interest

None declared.
